# Missed opportunities for family planning counselling among HIV-positive women receiving HIV Care in Uganda

**DOI:** 10.1186/s12905-020-00942-6

**Published:** 2020-05-05

**Authors:** Juliet Nabirye, Joseph K. B. Matovu, John Baptist Bwanika, Fredrick Makumbi, Rhoda K. Wanyenze

**Affiliations:** 1 Department of Disease Control and Environmental Health, Makerere University School of Public Health, P.O. Box 7072, Kampala, Uganda; 2 Busitema University Faculty of Health Sciences, Mbale, Uganda

**Keywords:** Contraceptives, Family planning counselling, HIV positive women, Postnatal, Antenatal care, Delivery

## Abstract

**Introduction:**

HIV-positive women who are still in the reproductive years need adequate sexual and reproductive health information to make informed reproductive health choices. However, many HIV-positive women who interface with the health system continue to miss out on this information. We sought to: a) determine the proportion of HIV-positive women enrolled in HIV care who missed family planning (FP) counselling; and b) assess if any association existed between receipt of FP counselling and current use of modern contraception to inform programming.

**Methods:**

Data were drawn from a quantitative national cross-sectional survey of 5198 HIV-positive women receiving HIV care at 245 HIV clinics in Uganda; conducted between August and November 2016. Family planning counselling was defined as provision of FP information (i.e. available FP methods and choices) to an HIV-positive woman by a health provider during ANC, at the time of delivery or at the PNC visit. Analyses on receipt of FP counselling were done on 2760 HIV-positive women aged 15–49 years who were not currently pregnant and did not intend to have children in the future. We used a modified Poisson regression model to determine the Prevalence Ratio (PR) as a measure of association between receipt of any FP counselling and current use of modern contraception, controlling for potential confounders. Analyses were performed using STATA statistical software, version 14.1.

**Results:**

Overall, 2104 (76.2%) HIV-positive women reported that they received FP counselling at any of the three critical time-points. Of the 24% (*n* = 656) who did not, 37.9% missed FP counselling at ANC; 41% missed FP counselling during delivery; while 54% missed FP counselling at the post-natal care visit. HIV-positive women who received any FP counselling were significantly more likely to report current use of modern contraception than those who did not (adjusted PR [adj. PR] = 1.21; 95% Confidence Interval [CI]: 1.10, 1.33).

**Conclusion:**

Nearly one-quarter of HIV-positive women did not receive any form of FP counselling when they interfaced with the healthcare system. This presents a missed opportunity for prevention of unintended pregnancies, and suggests a need for the integration of FP counselling into HIV care at all critical time-points.

## Introduction

Women of reproductive age account for about half of people living with Human Immunodeficiency Virus (HIV) globally [1]. Preventing unintended pregnancies among women living with HIV is one of the four comprehensive approaches for confronting unwanted pregnancy among People Living With HIV/AIDS (PLWHA), an approach that was adopted by the World Health Organization (WHO) to promote and prevent the transmission of HIV from mothers to their babies [2]. Provision of appropriate counselling and support as well as contraceptives to women living with HIV to meet their family planning needs is a cost-effective intervention to prevent mother-to-child transmission of HIV [3]. However, only a handful of women living with HIV have taken any credible steps to stop unplanned pregnancies partly because many women do not receive such information and support from the health providers.

In many African countries, many women are at risk for HIV infection and unintended pregnancy at the same time [4, 5]. For instance, in Uganda, women not only have high HIV prevalence [6] but also report high fertility levels [7]; with women living with HIV (WLHIV) reporting equally high levels of unintended pregnancy ranging from 51 to 84% [8, 9]. In Malawi, one study found that only 51.2% of WLHIV used some form of contraception [8], suggesting that up to 49% of WLHIV were at risk of getting unintended pregnancies. Similar findings were reported in another study conducted in Ethiopia which found a contraceptive prevalence rate of 30.2% [10], suggesting a high risk of unintended pregnancy. Indeed, a recent systematic review and meta-analysis of unintended pregnancy among WLHIV in sub-Saharan Africa found a pooled proportion of unintended pregnancy of 55.9% with the magnitude of unwanted and mistimed pregnancy in six studies ranging from 14 to 59% and 9 to 47.2%, respectively [11]. These studies show high rates of unwanted pregnancies for WLHIV implying that the unintended pregnancies could be substantially reduced if women used modern contraception.

Several reasons have been advanced to explain the low use of contraception among WLHIV including fears of the likely interaction between hormonal contraceptive methods and antiretroviral therapy [12], fear of side effects and the lack of social support [13]. Although there is evidence that receipt of family planning (FP) counselling can increase uptake of contraceptives [14], there are indications that many women go through the health system without receiving any form of FP counselling. For example, a study conducted among women attending antenatal care in Rwanda found that only 17% of pregnant women received any form of FP counselling on contraception [15]. In a study conducted among mothers in western Uganda, it was discovered that current users of FP methods could have been higher if village health teams counselled mothers and at the same time offered alternative contraceptive methods alternative contraceptive methods [16]. A similar study done among HIV positive women in Nigeria found that although women had knowledge of contraceptives, the percentage of women using any form of contraception was as low as 36% [17]. Collectively, these studies suggest that few women receive FP counselling, resulting in very low use of contraceptive services among pregnant women. However, most of these studies were conducted among pregnant women in the general population and not among WLHIV in particular. Besides, only a handful of studies have sought to determine what proportion of WLHIV miss FP counselling when they interface with the healthcare system. This study sought to: a) determine the proportion of WLHIV who missed the opportunity to receive FP counselling, and b) assess the effect of receiving FP counselling on current use of modern contraceptives in order to inform the provision of FP counselling to pregnant WLHIV who access health services during antenatal care, delivery or postnatal care.

## Methods

Data were drawn from a quantitative national cross-sectional survey of WLHIV receiving HIV care at 245 HIV clinics in Uganda. The survey objectives were to: a) assess the unmet need for FP and b) determine the uptake of other reproductive health services including cervical cancer screening [18]. Questions relating to pregnancy history, use of FP services, and whether or not women had ever received any form of FP counselling were administered to all respondents who consented to participate in the survey using interviewer-administered questionnaires. Women who reported that they had ever given birth were asked if they were currently pregnant, and if so, whether or not they wanted to get pregnant within the past two years preceding their pregnancy or to delay the pregnancy for two or more years. Similarly, women who were not currently pregnant (but who had ever been pregnant) were asked if they wanted to become pregnant with their most recent pregnancy during the two years preceding the pregnancy or to delay the pregnancy for two or more years. Women who were pregnant at the time of interview or whose last pregnancy was before this interview but who wanted to delay their pregnancy for two or more years were termed as those with ‘intention to become pregnant in the future ’.

### Study sites

Data for the primary survey were collected at 245 public and private HIV clinics across five geographical regions in Uganda (Central, Northern, Eastern, Western, and Kampala). Kampala, which is the capital city of Uganda, was considered as a separate region owing to its uniqueness. The health facilities were selected from public and private facilities across several levels of service delivery; hospitals and lower level health centers (HCIV, HCIII, and HCII) that had HIV care clinics and had a minimum patient volume that was considered for their eligibility to be in the sampling frame.

### Survey measures

This analysis was restricted to WLHIV who were not currently pregnant and who did not want to become pregnant in the future. We obtained data on socio-demographic characteristics, disclosure of HIV status to sexual partner, relationship status, use of antenatal care (ANC) services and delivery at a health facility; receipt of post-natal services, prior and current use of modern contraception and pregnancy history. Women were asked if they received any form of FP counselling when they interfaced with the health facility, and if they responded in the affirmative, they were asked at what point they received FP counselling, to determine if it was during ANC, immediately after delivery or during post-natal care. Women were also asked if they were currently using any of the following FP methods: pills, injectable, implant, intra-uterine device, female sterilization, vasectomy (by their male partners) or condom use for FP purposes. Women were considered to have received FP counselling if, at ANC, during delivery or post-natal care, they reported that they received information about FP methods and choices and/or discussed FP issues with a health provider. A missed opportunity for FP counselling was defined as a woman’s clinic visit at which a staff member at the health facility did not provide any FP counselling. Women who reported that they did not receive such information or discussion on FP at any of the three visits were considered to have a missed opportunity for FP.

We used responses on household possessions to create an index representing a wealth proxy for the respondents interviewed. The list of household assets probed for included a radio, television set, bicycle, motorcycle, own/family home, cell phone, regular (landline) phone, computer, income generating business, indoor bathroom, running water either inside the house or inside the compound of your house, electricity, car, generator and solar electricity. To construct the socio-economic status (SES) index, each household item was assigned a weight ascertained through principal components analysis. Then, the scores were standardized in relation to a standard normal distribution with a mean of zero and a standard deviation of one. The scores on household possessions were then summed up and individuals were ranked and sub-divided into wealth quintiles, depending on their scores, with each quintile containing 20% of the participants. The SES index was divided into five categories, namely: Lowest, second, middle, fourth and highest wealth quintile.

### Data analysis

Analyses were done on 2760 women to provide descriptive statistics for women’s socio-demographic characteristics. The key variables of FP counselling were determined as proportions. The primary outcome was receiving FP counselling at ANC, delivery or at post-natal care (PNC). Receipt of FP counselling was determined by women’s socio-demographic characteristics. To determine the association between receipt of FP counselling and current use of modern contraception, we used prevalence ratio (PR) as the measure of association. Condom use was excluded in the final model because it tends to overestimate the modern contraception uptake and it is not reliable as a method of contraception if not used consistently and correctly.

## Results

### Respondents’ characteristics

Table 1 shows the socio-demographic characteristics of the 2760 women who were included in the analysis. Nearly half (46%, *n* = 1284) were aged 30–39 years, with the Eastern region having slightly more than half of the women (52.1%) aged 30–39 years. More than half of the respondents (58%, *n* = 1600) were married, with the highest proportion of married HIV-positive women reported in the Eastern region (66.7%) followed by the Western region (63.1%) and Kampala region (58%) in that order. About a quarter of the women (24.3%, *n* = 670) were in a relationship but not married, with the highest proportion reported in the Central region (36.4%), Kampala region (32%) and the Northern region (27.3%) in that order. More than half of the women (57.4%, *n* = 1585) had primary education while 24% (*n* = 656) had secondary education with the Western region (65.1%), the Central region (60.4%) and the Northern region (59%) having the highest proportion of women with primary education. It is important to note that nearly a quarter (24.6%) of women in the Northern region had no education.

**Table 1:** Socio-demographic characteristics of 2760 HIV-positive women enrolled in the main survey

		Region				
Characteristic	Total, ***N*** = 2760 (%)	Kampala, ***N*** = 525 (%)	Central, ***N*** = 533 (%)	Eastern, ***N*** = 574 (%)	Western, ***N*** = 550 (%)	Northern, ***N*** = 578 (%)
**Age**						
15–24	240 (8.7)	71 (13.5)	45 (8.4)	32 (5.6)	43 (7.8)	49 (8.5)
25–29	420 (15.2)	105 (20)	77 (14.4)	73 (12.7)	86 (15.6)	79 (13.7)
30–39	1284 (46.5)	239 (45.5)	252 (47.3)	299 (52.1)	255 (46.4)	239 (41.3)
40–49	816 (29.6)	110 (21)	159 (29.8)	170 (29.6)	166 (30.2)	211 (36.5)
**Health Facility of Enrolment**						
Hospital	810 (29.4)	97 (18.5)	163 (30.8)	159 (27.7)	178 (32.5)	213 (36.9)
HC IV	850 (30.9)	47 (9.0)	140 (26.4)	234 (40.8)	213 (38.9)	216 (37.4)
HC III	838 (30.4)	228 (43.4)	195 (36.8)	137 (23.9)	139 (25.4)	139 (24.0)
HC II	200 (7.3)	139 (26.5)	1 (0.2)	37 (6.4)	18 (3.3)	5 (0.9)
Private health unit	45 (1.6)	14 (2.7)	24 (4.5)	7 (1.2)	0 (0.0)	0 (0.0)
Others	12 (0.4)	0 (0.0)	7 (1.3)	0 (0.0)	0 (0.0)	5 (0.9)
**Religion**						
Catholic	1159 (42.0)	178 (33.9)	233 (43.7)	171 (29.8)	230 (41.8)	347 (60.0)
Anglican / Protestant	874 (31.7)	141 (26.9)	156 (29.3)	223 (38.9)	212 (38.5)	142 (24.6)
Moslem	323 (11.7)	101 (19.2)	72 (13.5)	85 (14.8)	22 (4.0)	43 (7.4)
Pentecostal / Born Again / Evangelical	339 (12.3)	92 (17.5)	56 (10.5)	90 (15.7)	61 (11.1)	40 (6.9)
Others Religions	57 (2.3)	12 (2.3)	15 (2.8)	5 (0.9)	20 (3.6)	5 (0.9)
**Marital status**						
Never married	39 (1.4)	8 (1.5)	1 (0.2)	8 (1.4)	9 (1.6)	13 (2.2)
In relationship but not married	670 (24.3)	168 (32.0)	194 (36.4)	77 (13.4)	73 (13.3)	158 (27.3)
Married	1600 (58.0)	301 (57.3)	258 (48.4)	383 (66.7)	347 (63.1)	311 (53.8)
Divorced/separated	264 (9.6)	36 (6.9)	52 (9.8)	54 (9.4)	68 (12.4)	54 (9.3)
Widowed	187 (6.8)	12 (2.3)	28 (5.3)	52 (9.1)	53 (9.6)	42 (7.3)
**Education**^**a**^						
No education	453 (16.4)	40 (7.6)	85 (15.9)	87 (15.2)	99 (18.0)	142 (24.6)
Primary	1585 (57.4)	241 (45.9)	322 (60.4)	323 (56.3)	358 (65.1)	341 (59)
Secondary	656 (23.8)	208 (39.6)	119 (22.3)	160 (27.9)	91 (16.5)	78 (13.5)
More than secondary	58 (2.1)	36 (6.9)	5 (0.9)	4 (0.7)	1 (0.2)	12 (2.1)
Missing	8 (0.3)	0 (0.0)	2 (0.4)	0 (0.0)	1 (0.2)	5 (0.9)
**Wealth quintile**						
Lowest	623 (22.6)	13 (2.5)	105 (19.7)	153 (26.7)	141 (25.6)	211 (36.5)
Second	557 (20.2)	23 (4.4)	112 (21.0)	142 (24.7)	115 (20.9)	165 (28.5)
Middle	537 (19.5)	38 (7.2)	107 (20.1)	147 (25.6)	129 (23.5)	116 (20.1)
Fourth	541 (19.6)	171 (32.6)	119 (22.3)	84 (14.6)	112 (20.4)	55 (9.5)
Highest	502 (18.2)	280 (53.3)	90 (16.9)	48 (8.4)	53 (9.6)	31 (5.4)
**Owns a radio**						
No	1035 (37.5)	191 (36.4)	157 (29.5)	227 (39.5)	203 (36.9)	257 (44.5)
Yes	1725 (62.5)	334 (63.6)	376 (70.5)	347 (60.5)	347 (63.1)	321 (55.5)
**Owns a Television**						
No	2092 (75.8)	182 (34.7)	404 (75.8)	501 (87.3)	477 (86.7)	528 (91.3)
Yes	668 (24.2)	343 (65.3)	129 (24.2)	73 (12.7)	73 (13.3)	50 (8.7)
**Owns a Cell phone**						
No	559 (20.3)	38 (7.2)	95 (17.8)	138 (24.0)	121 (22.0)	167 (28.9)
Yes	2201 (79.7)	487 (92.8)	438 (82.2)	436 (76.0)	429 (78.0)	411 (71.1)
**Proximity (in Km) to health facility**						
1–4	1122 (41.0)	267 (51.0)	227 (42.7)	233 (40.8)	179 (32.8)	216 (38.2)
5–9	653 (23.8)	108 (20.6)	120 (22.6)	137 (24.0)	116 (21.2)	172 (30.4)
10–15	330 (12.1)	57 (10.9)	47 (8.9)	58 (10.2)	90 (16.5)	78 (13.8)
15+	633 (23.1)	92 (17.6)	137 (25.8)	143 (25)	161 (29.5)	100 (17.7)
**On antiretroviral therapy**						
No	82 (3.0)	27 (5.2)	0 (0.0)	10 (1.7)	27 (4.9)	18 (3.1)
Yes	2667 (97.0)	497 (94.8)	529 (100.0)	563 (98.3)	523 (95.1)	555 (96.9)
**Duration on ART (Years)**						
Not on ART	251 (9.5)	83 (16.8)	57 (10.8)	39 (7)	39 (7.5)	33 (6.0)
< 1 year	281 (10.6)	56 (11.3)	64 (12.1)	54 (9.7)	52 (10.0)	55 (10.1)
< 2 years	345 (13.0)	71 (14.4)	70 (13.3)	59 (10.6)	71 (13.7)	74 (13.5)
2+ years	1767 (66.8)	284 (57.5)	336 (63.8)	405 (72.7)	357 (68.8)	385 (70.4)
**Disclosed HIV status to partner**						
No	2313 (84.4)	367 (70.6)	414 (78.0)	504 (88.3)	479 (87.6)	549 (96.3)
Yes	426 (15.6)	153 (29.4)	117 (22.0)	67 (11.7)	68 (12.4)	21 (3.7)
**Number of biological children**						
0	724 (26.2)	124 (23.6)	175 (32.8)	124 (21.6)	175 (31.8)	126 (21.8)
1	135 (4.9)	58 (11.0)	21 (3.9)	17 (3.0)	17 (3.1)	22 (3.8)
2	320 (11.6)	101 (19.2)	63 (11.8)	44 (7.7)	60 (10.9)	52 (9.0)
3	400 (14.5)	98 (18.7)	63 (11.8)	83 (14.5)	81 (14.7)	75 (13.0)
4+	1181 (42.8)	144 (27.4)	211 (39.6)	306 (53.3)	217 (39.5)	303 (52.4)
**Received any FP Counselling**						
No	656 (23.8)	154 (29.3)	145 (27.2)	118 (20.6)	100 (18.2)	139 (24.0)
Yes	2104 (76.2)	371 (70.7)	388 (72.8)	456 (79.4)	450 (81.8)	439 (76.0)
**Number of FP Counselling Visits**						
0	656 (23.8)	154 (29.3)	145 (27.2)	118 (20.6)	100 (18.2)	139 (24.0)
1	502 (18.2)	118 (22.5)	137 (25.7)	56 (9.8)	77 (14.0)	114 (19.7)
2	688 (24.9)	134 (25.5)	156 (29.3)	121 (21.1)	113 (20.5)	164 (28.4)
3	914 (33.1)	119 (22.7)	95 (17.8)	279 (48.6)	260 (47.3)	161 (27.9)

^a^ Education categories refer to the highest level of education attended, whether or not that level was completed

About 43% (*n* = 1180) of women were in the lowest or second lowest wealth quintile with women in the Northern region (65%), those in the Eastern region (51.4%) and those in the Western region of Uganda (46.5%) more likely to be in the lowest or second lowest wealth quintile than women in other regions. More than half of women in Kampala region (53.3%) were in the highest wealth quintile while only a small proportion of women in the other regions (5.4–16.9%) were in this category.

Forty-one per cent (*n* = 1122) of the respondents lived within less than 4 km to a health facility in all the regions with Kampala having more than half of the respondents living within this distance to a health facility. Majority (97%, *n* = 2667) of the respondents were on antiretroviral therapy (ART) with slightly more than two-thirds (66.8%) having been on ART for more than two years. The Central region (100%), Eastern region (98.3%) and the Northern region (96.9%) recorded the highest proportion of HIV-positive women on ART. Only 15.6% (*n* = 426) of HIV-positive women reported that they disclosed their HIV sero-positive status to their sexual partners, and this trend was observed across all the other regions. Forty-three per cent (*n* = 1181) of women had four or more (4+) biological children, with the proportion of those reporting 4+ biological children recorded in the Eastern (53.3%) and the Northern regions (52.4%) while Kampala region (27.4%) had the lowest proportion of women with 4+ biological children.

### Receipt of FP counselling

Table 2 shows the percentage of women in HIV care that were not currently pregnant and who did not want to become pregnant in the future, stratified by whether or not they received any FP counselling; and if so, whether or not they received FP counselling during ANC visit, at the time of delivery or at the post-natal care (PNC) visit. Slightly more than three quarters (76%) of the women reported that they received FP counselling at any of the three points of care (ANC, delivery and PNC). Receipt of FP counselling was highest at ANC (62.1%, *n* = 1715) but was slightly lower at the time of delivery (59%, *n* = 1629) and at the PNC visit (46.2%, *n* = 1276). By region, the proportion of women who received FP counselling was highest in the Western region (81.8%), the Eastern (79.4%) and the Northern region (76%) but was lowest in the Central region (72.8%) and Kampala (70.8%) (Table 1). Approx. 58% of the women were found to have received FP counselling at two (24.9%) or three visits (33.1%), with wide variations across regions (Table 1).

**Table 2:** Percentage of women in HIV care who are not currently pregnant and would not like to become pregnant that received FP counselling overall; during ANC visit, during delivery or during Post-Natal Care

Characteristic	***N*** = 2760			
	ANC	Delivery	Post-natal Care	Any FP counselling
Total	**1715 (62.1)**	**1629 (59.0)**	**1276 (46.2)**	**2104 (76.2)**
**Age-group**				
15–24	138 (57.5)	134 (55.8)	106 (44.2)	172 (71.7)
25–29	259 (61.7)	267 (63.6)	206 (49.0)	328 (78.1)
30–39	822 (64.0)	779 (60.7)	623 (48.5)	1001 (78.0)
40–49	496 (60.8)	449 (55.0)	341 (41.8)	603 (73.9)
**Region**				
Kampala	278 (53.0)	280 (53.3)	185 (35.2)	371 (70.7)
Central	268 (50.3)	310 (58.2)	156 (29.3)	388 (72.8)
Eastern	420 (73.2)	362 (63.1)	353 (61.5)	456 (79.4)
Western	365 (66.4)	365 (66.4)	353 (64.2)	450 (81.8)
Northern	384 (66.4)	312 (54.0)	229 (39.6)	439 (76.0)
**Enrolment Health Facility**				
Hospital	479 (59.1)	446 (55.1)	349 (43.1)	592 (73.1)
HC IV	578 (68.0)	543 (63.9)	466 (54.8)	678 (79.8)
HC III	510 (60.9)	499 (59.5)	354 (42.2)	651 (77.7)
HC II	111 (55.5)	104 (52.0)	74 (37.0)	135 (67.5)
Private health unit	28 (62.2)	29 (64.4)	29 (64.4)	37 (82.2)
Others	6 (50.0)	5 (41.7)	2 (16.7)	7 (58.3)
**Religion**				
Catholic	718 (61.9)	670 (57.8)	524 (45.2)	881 (76.0)
Anglican / Protestant	545 (62.4)	528 (60.4)	434 (49.7)	676 (77.3)
Moslem	208 (64.4)	204 (63.2)	142 (44.0)	248 (76.8)
Pentecostal	208 (61.4)	196 (57.8)	156 (46.0)	256 (75.5)
Others	33 (57.9)	28 (49.1)	18 (31.6)	39 (68.4)
**Marital status**				
Never married	21 (53.8)	21 (53.8)	16 (41.0)	27 (69.2)
In relationship but not married	371 (55.4)	356 (53.1)	231 (34.5)	464 (69.3)
Married	1067 (66.7)	1001 (62.6)	836 (52.3)	1277 (79.8)
Divorced/separated	154 (58.3)	160 (60.6)	119 (45.1)	209 (79.2)
Widowed	102 (54.5)	91 (48.7)	74 (39.6)	127 (67.9)
**Education**				
No education	263 (58.1)	241 (53.2)	203 (44.8)	325 (71.7)
Primary	987 (62.3)	943 (59.5)	734 (46.3)	1219 (76.9)
Secondary	428 (65.2)	406 (61.9)	314 (47.9)	516 (78.7)
More than secondary	35 (60.3)	37 (63.8)	24 (41.4)	41 (70.7)
Missing	2 (25.0)	2 (25.0)	1 (12.5)	3 (37.5)
**Wealth quintile**				
Lowest	408 (65.5)	352 (56.5)	306 (49.1)	487 (78.2)
Second	379 (68,0)	324 (58.2)	270 (48.5)	427 (76.7)
Middle	316 (58.8)	332 (61.8)	265 (49.3)	410 (76.4)
Fourth	329 (60.8)	324 (59.9)	237 (43.8)	413 (76.3)
Highest	283 (56.4)	297 (59.2)	198 (39.4)	367 (73.1)
**On antiretroviral therapy**				
No	49 (59.8)	36 (43.9)	37 (45.1)	56 (68.3)
Yes	1661 (62.3)	1588 (59.5)	1235 (46.3)	2041 (76.5)
**Duration on ART (Years)**^a^				
Not on ART	125 (49.8)	122 (48.6)	89 (35.5)	175 (69.7)
< 1 year	154 (54.8)	144 (51.2)	116 (41.3)	201 (71.5)
< 2 years	224 (64.9)	204 (59.1)	164 (47.5)	268 (77.7)
2+ years	1145 (64.8)	1106 (62.6)	857 (48.5)	1382 (78.2)
**Disclosed HIV status to partner**				
No	1499 (64.8)	1403 (60.7)	1130 (48.9)	1798 (77.7)
Yes	200 (46.9)	211 (49.5)	136 (31.9)	287 (67.4)
**Number of biological children**				
0	387 (53.5)	382 (52.8)	289 (39.9)	505 (69.8)
1	65 (48.1)	63 (46.7)	42 (31.1)	88 (65.2)
2	201 (62.8)	193 (60.3)	154 (48.1)	254 (79.4)
3	256 (64)	245 (61.3)	181 (45.3)	309 (77.3)
4+	806 (68.2)	746 (63.2)	610 (51.7)	948 (80.3)

^a^Expressed out of those who reported that they were on ART

Table 2 also shows that receipt of FP counselling differed by age, region of residence, point of delivery, health facility where women were enrolled, and ART status. At least 60% of all women across all age groups received FP counselling during ANC with the highest proportion recorded among those aged 30 to 39 years. At the time of delivery, women aged 25–29 were the highest recipients of FP counselling at nearly 64%. Younger women below 24 years and women aged 40 or more years were the lowest recipients of FP counselling at post-natal care. The Western region had the highest proportion of women who received any form of FP counselling at 82%. Kampala and Central region had the lowest proportion of women receiving FP counselling during ANC with nearly half of women reporting that they did not receive any form of FP counselling during contact with a healthcare provider.

At the point of delivery, the Northern region and Kampala recorded at least half of the women receiving FP counselling while the other half missed out on this service. During postnatal care, the Northern region registered the lowest proportion of women who received FP counselling at 40%. Provision of FP counselling was highest at health Centre IIs and IVs with approximately 80% of the women receiving FP counselling at these facilities. A higher proportion of married women and women educated up to secondary level received FP counselling across the three points of care than unmarried and divorced women although PNC was the least used point for FP counselling. Women on ART were beneficiaries of FP counselling across the three levels of care with 62% of them obtaining FP counselling at ANC, 60% during delivery and 46% at postnatal care. A higher proportion of women (80% or higher) who had been on ART for two years and those with four or more biological children received FP counselling more than their counterparts across the three points of care.

### Association between receipt of any FP counselling and current use of modern contraception

Table 3 shows the association between receipt of any FP counselling and current use of modern contraception among women who were not pregnant and who did not want to have any other children in the future. Overall, current use of a modern contraceptive method was 21% higher among women who received any FP counselling compared to those who did not (adjusted [adj.] PR: 1.21; 95% confidence interval [CI]: 1.10, 1.33). Current use of modern contraception was also 28% higher among women who had attained more than secondary level of education compared to those with primary level of education and 28% higher among those who had spent more than two years on ART. Current use of modern contraceptive use was also more than 30% higher among women who had two or more biological children than their counterparts. We found that current use of modern contraception increased with the increasing number of FP counselling visits but this analysis was restricted to the bivariate analysis due to collinearity between ‘any FP counselling’, the primary outcome, and ‘number of FP counselling visits’.

**Table 3:** Factors associated with receipt of any FP Counselling and current use of modern contraception (excluding condom use)

	Bivariate		Multivariable	
Background characteristic	PR (95% CI)	***p***-value	PR (95% CI)	p-value
**Received any FP counselling**				
No	Ref		Ref	
Yes	1.31 (1.19, 1.44)	< 0.001	1.21 (1.10, 1.33)	< 0.001*
**Number of FP counselling visits**				
None	Ref			
1	1.14 (1.01, 1.29)	0.04		
2	1.30 (1.62, 1.45)	< 0.001		
3	1.40 (1.27, 1.55)	< 0.001		
**Age**				
15–24	Ref		Ref	
25–29	1.08 (0.94,1.24)	0.30	1.00 (0.86,1.15)	0.97
30–39	1.11 (0.98,1.25)	0.11	0.98 (0.85,1.12)	0.73
40–49	0.74 (0.64,0.86)	0.00	0.67 (0.58,0.79)	0.00
**Region**				
Northern	Ref		Ref	
Kampala	1.29 (1.14,1.46)	0.00	1.29 (1.11,1.49)	**0.00**
Central	1.19 (1.05,1.36)	0.01	1.21 (1.06,1.37)	**0.00**
Eastern	1.49 (1.33,1.66)	0.00	1.41 (1.25,1.58)	**0.00**
Western	1.35 (1.20,1.52)	0.00	1.31 (1.16,1.48)	**0.00**
**Level Health Facility**				
Hospital	Ref		Ref	
Health Center IV	1.13 (1.04,1.24)	0.01	1.09 (0.99,1.19)	0.07*
Health Center III	1.08 (0.99,1.19)	0.09	1.05 (0.96,1.15)	0.28
Health Center II	1.16 (1.01,1.33)	0.03	1.05 (0.90,1.22)	0.53
Private Health Unit	0.94 (0.68,1.29)	0.68	0.88 (0.64,1.21)	0.44
Others	1.00 (0.57,1.77)	0.99	1.25 (0.72,2.17)	0.42
**Religion**				
Catholic	Ref			
Anglican / Protestant	1.09 (1.01,1.18)	0.03	1.02 (0.94,1.11)	0.59
Moslem	1.03 (0.92,1.15)	0.63	0.96 (0.85,1.08)	0.47
Protestant / Born Again	0.97 (0.86,1.09)	0.60	0.91 (0.81,1.03)	0.13
Other Religions	0.94 (0.72,1.23)	0.64	0.87 (0.66,1.14)	0.32
**Marital status**				
Never married	Ref		Ref	
In relationship but not married	1.77 (1.07,2.94)	0.03	1.38 (0.82,2.34)	0.23
Married	2.11 (1.28,3.49)	0.00	1.43 (0.84,2.44)	0.19
Divorced/separated	1.63 (0.97,2.73)	0.07	1.50 (0.89,2.53)	0.13
Widowed	1.23 (0.72,2.11)	0.45	1.28 (0.74,2.21)	0.38
**Education**				
No education	Ref		Ref	
Primary	1.09 (0.98,1.21)	0.095	1.05 (0.94,1.16)	0.39
Secondary	1.13 (1.01,1.27)	0.036	1.07 (0.95,1.20)	0.27
Above Secondary	1.22 (0.97,1.53)	0.088	1.28 (1.00,1.63)	0.05*
**Wealth quintile**				
Lowest	Ref		Ref	
Second	1.14 (1.02,1.27)	0.02	1.11 (0.99,1.24)	0.06
Middle	1.19 (1.07,1.33)	0.00	1.12 (1.01,1.25)	0.04*
Fourth	1.15 (1.03,1.29)	0.01	1.08 (0.96,1.21)	0.22
Highest	1.12 (1.0,1.25)	0.06	1.05 (0.92,1.19)	0.49
**Duration on ART**				
Not on ART	Ref			
< 1 year	1.11 (0.92,1.33)	0.27	1.10 (0.92,1.32)	0.30
< 2 years	1.29 (1.10,1.52)	0.00	1.28 (1.09,1.51)	0.00*
2+ years	1.23 (1.07,1.43)	0.00	1.28 (1.10,1.48)	0.00
**Number of biological children**				
0	Ref		Ref	
1	1.18 (0.97,1.43)	0.10	1.09 (0.86,1.37)	0.49
2	1.47 (1.29,1.66)	0.00	1.30 (1.08,1.57)	0.01*
3	1.51 (1.34,1.7)	0.00 0.00	1.37 (1.14,1.65)	0.00
4+	1.42 (1.28,1.57)	0.00	1.38 (1.16,1.66)	0.00

## Discussion

Our study of missed opportunities for FP counselling among HIV-positive women receiving HIV care in Uganda highlights two important findings: a) up to 24% of HIV-positive women who interfaced with the healthcare system when they were pregnant, at the time of delivery or at the post-natal care visit did not receive any form of FP counselling; and b) receipt of any FP counselling at any of the three time points is associated with current use of modern contraception. We found that receipt of FP counselling was higher during ANC (62.1%), declined at the time of delivery (59%) and was lowest at the post-natal care visit (46.2%). These findings imply that 38% of women missed FP counselling at ANC; 41% missed FP counselling at the time of delivery, while 54% missed FP counselling at the post-natal care visit. Similar findings have been reported elsewhere [19, 20]. Venkataramani et al. found that only 0.3% of parents were counseled about FP during 4261 preventive care visits [19] while Moore et al. found that 61% of all postpartum women across the 21 countries studied had an unmet need for FP [20]. Collectively, our findings highlight a missed opportunity for FP counselling among HIV-positive women who have an unmet need for limiting childbirth and suggest a need for full-scale integration of FP counselling at all critical time-points, but most importantly at the post-natal care visit.

The fact that FP counselling was highest at ANC but lowest during post-natal services raises serious public health concerns. It shows that a significant proportion of HIV positive women who interface with the health system at the post-natal care visit are not provided with FP counselling yet they are at an increased risk for unwanted and short-interval pregnancies [21]. Although fewer women attend post-natal care services when compared to those who attend ANC [22], possibly due to a perception of having received sufficient information about childcare and the management of post-natal complications at ANC [23, 24], this small number of women should still be targeted with adequate FP information when they come for post-natal care services. Our findings point to the fact that the more women interface with the health system, the more they are likely to obtain FP counselling. This is similar to a study done in Pakistan, where the use of contraception increased with the number of ANC visits from 8% among women who did not make any antenatal visit to 32% of women who made four ANC visits [25].

Our findings have implications for the provision of contraceptive services to pregnant women across countries. For instance, a study of post-partum contraception among 250 women in Edinburg found that nearly all women (96.7%) who attended post-natal care services did not want to have a baby in the following year but up to 35.2% of them did not know what contraception to use post-natal [26]. Indeed, evidence shows that a significant proportion of women are willing to use contraception post-natal only that they lack the information needed to make informed contraceptive decisions [26, 27]. Thus, the lack of FP counselling, acts as a missed opportunity to prevent unintended pregnancy and short-interval pregnancies. The low provision of FP counselling to women during delivery may not be surprising given that at that time, the woman is more concerned about safe delivery or the need to return home following a safe delivery than issues of child spacing or limiting childbirth. This observation is in agreement with previous studies that show that contraceptive counselling during delivery may not be very effective because women are in a hurry to go home [16].

The finding that FP counselling was associated with current use of modern contraceptive methods is in agreement with previous studies in which women that received FP messages from a health worker had higher odds of using contraceptives than their counterparts and suggest an opportunity to reduce the unmet need for FP services through counselling [28, 29]. Studies also show that in programs that focus on HIV care and treatment, adherence counselling offers an exceptional chance to address preventive health recommendations, including FP [29]. The continued use of modern FP methods by HIV-infected women subsequently prevents birth of HIV-positive infants and reduces cost of Preventing Mother-To-Child Transmission (PMTCT) in addition to ensuring spaced pregnancies, which results in healthier babies, despite the mother’s HIV status [30].

### Strengths and limitations of the study

Our study had several limitations. For instance, although we found an association between FP counselling and current use of modern contraception, we cannot infer a causal relationship between the two. Precisely, this study cannot depict that receiving FP counselling always results in uptake of contraception due to its cross-sectional nature. We also recognize that HIV-positive women who came for ANC services could have been different from other HIV-positive women who did not utilize ANC services. This affects the generalizability of the study findings to all HIV-positive women in Uganda. Besides, while we collected qualitative data about the barriers to uptake of contraceptive services among HIV-positive women as part of this study, these results were not yet available at the time of writing this paper. Therefore, we cannot fully explain why some women did not use contraception services even after receiving FP counselling. However, as our study findings show, receipt of FP counselling was associated with higher odds of current use of modern contraception suggesting that FP counselling is a key determinant of current contraceptive use among HIV-positive women. The above-mentioned limitations notwithstanding, our study addressed one of the key areas, which remains salient in the effort needed to prevent mother-to-child transmission of HIV. It shows that there is a missed opportunity for FP counselling among HIV-positive women who interface with the healthcare system, calling for a need to integrate FP counselling into HIV care at critical times during antenatal care, during delivery and when women return for post-natal services to reduce unmet need for FP among HIV-positive women.

## Conclusion

In conclusion, our study shows that nearly a quarter of HIV-positive women who interface with the healthcare system while pregnant, during delivery or at the post-natal care visit miss the opportunity to be counseled about contraception, which presents a missed opportunity for prevention of unwanted and untimed pregnancies. This missed opportunity becomes even more prominent when we realize that women who received FP counselling at any of the three critical time-points were significantly more likely to report current use of modern contraception than women who did not. Given the increasing popularity of hormonal contraception methods, efforts should be directed at counselling women about the importance of contraception to avoid unwanted pregnancies and reduce mother-to-child transmission of HIV and there is a need to enhance FP integration into HIV care in order to increase access to contraceptives among HIV-positive women in care. We particularly recommend an improvement on provision of FP counselling to HIV-positive women who attend the postnatal care visit since it improves uptake of modern contraceptives.

## Data Availability

Data for this study is available upon reasonable request from the corresponding author.

## Abbreviations

ANC: Antenatal Care
ART: Anti-Retroviral Therapy
FP: Family Planning
HIV: Human Immunodeficiency
PLWHIV: People Living With HIV
PMTCT: Prevention of Mother to Child Transmission
PR: Prevalence Ratio
WLHIV: Women Living with HIV
